# Detecting Hachimoji DNA: An Eight-Building-Block Genetic System with MoS_2_ and Janus MoSSe Monolayers

**DOI:** 10.1021/acsami.3c18400

**Published:** 2024-04-18

**Authors:** Vasudeo Babar, Sitansh Sharma, Abdul Rajjak Shaikh, Romina Oliva, Mohit Chawla, Luigi Cavallo

**Affiliations:** † Physical Sciences and Engineering Division, KAUST Catalysis Center, King Abdullah University of Science and Technology (KAUST), Thuwal 23955-6900, Saudi Arabia; ‡ Department of Research and Innovation, STEMskills Research and Education Lab Private Limited, Faridabad, Haryana 121002, India; § Department of Sciences and Technologies, University Parthenope of Naples, Centro Direzionale Isola C4, 80143 Naples, Italy

**Keywords:** 2D-materials, DNA sensing, density functional theory, MoS_2_, MoSSe

## Abstract

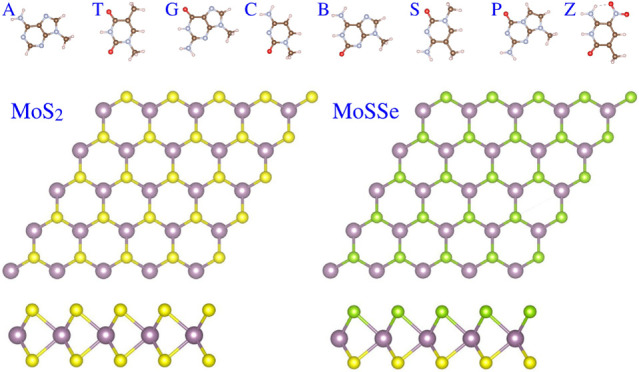

In the pursuit of personalized medicine, the development of efficient, cost-effective, and reliable DNA sequencing technology is crucial. Nanotechnology, particularly the exploration of two-dimensional materials, has opened different avenues for DNA nucleobase detection, owing to their impressive surface-to-volume ratio. This study employs density functional theory with van der Waals corrections to methodically scrutinize the adsorption behavior and electronic band structure properties of a DNA system composed of eight hachimoji nucleotide letters adsorbed on both MoS_2_ and MoSSe monolayers. Through a comprehensive conformational search, we pinpoint the most favorable adsorption sites, quantifying their adsorption energies and charge transfer properties. The analysis of electronic band structure unveils the emergence of flat bands in close proximity to the Fermi level post-adsorption, a departure from the pristine MoS_2_ and MoSSe monolayers. Furthermore, leveraging the nonequilibrium Green’s function approach, we compute the current–voltage characteristics, providing valuable insights into the electronic transport properties of the system. All hachimoji bases exhibit physisorption with a horizontal orientation on both monolayers. Notably, base G demonstrates high sensitivity on both substrates. The obtained current–voltage (*I*–*V*) characteristics, both without and with base adsorption on MoS_2_ and the Se side of MoSSe, affirm excellent sensing performance. This research significantly advances our understanding of potential DNA sensing platforms and their electronic characteristics, thereby propelling the endeavor for personalized medicine through enhanced DNA sequencing technologies.

## 1 Introduction

Biomolecular detection holds significant importance in disease diagnostics and biomolecular analysis, driving the pursuit of rapid, sensitive, and selective detection methods for deoxyribonucleic acid (DNA) and other small biomolecules.^1,2^ In addition to the four natural bases—adenine (A), thymine (T), guanine (G), and cytosine (C), DNA may contain modified bases in small quantities.^3^ DNA base modification serves as a critical epigenetic mechanism regulating gene expression in both plants and animals.

**Figure 1:**
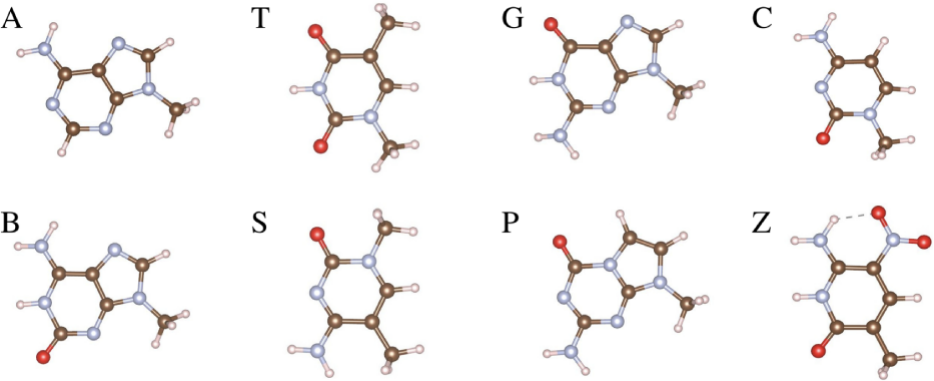
Molecular structures of hachimoji natural and modified DNA bases. In the representation, C, N, O, and H atoms are denoted by brown, gray, red, and pink balls, respectively.

In recent years, the field of synthetic biology has made significant progress in expanding the genetic code of DNA through the development of novel modified nucleotides. These specialized nucleotides have been extensively investigated for various applications, including precise site-specific labeling,^4−6^ targeted detection probing,^7,8^ and structural analysis of nucleic acids.^9−17^ In this scenario, Hoshika et al.^18^ integrated modified nucleotides (B, S, P, and Z) with the natural bases (A, T, G, and C) into oligonucleotides. Specifically, B represents 6-amino-9-(1′-β-d-2′-deoxyribofuranosyl)-4-hydroxy-5-(hydroxymethyl)-oxolan-2-yl]-1*H*-purin-2-one, S is 3-methyl-6-amino-5-(1′-β-d-2′-deoxyribofuranosyl)-pyrimidin-2-one, P is 2-amino-8-(1′-β-d-2′-deoxyribofuranosyl)-imidazo-[1,2*a*]-1,3,5-triazin-[8*H*]-4-one, and Z is 6-amino- 3-(1′-β-d-2′-deoxyribofuranosyl)-5-nitro-1*H*-pyridin-2-one. A, T, G, and C represent adenine, thymine, guanine, and cytosine, respectively. This ground breaking development resulted in the creation of DNA containing an expanded eight-base genetic alphabet (A, T, G, C, B, S, P, and Z, as illustrated in Figure 1). This innovative DNA structure exhibits significantly enhanced information-storage capacity compared to the conventional four-base natural alphabet, as well as previously reported six-base systems, which relied on combinations of canonical pairs with either Romesberg’s hydrophobic pairs^19^ or Benner’s P:Z pair.^20^ This newly engineered class of nucleic acids has been named “hachimoji” (eight-letter) DNA.

One potential application of an expanded genetic code is diagnostic in medicine. Optical and electrical detection methods using fluorescent or electrochemical labels are commonly employed but involve complex and costly procedures compared to label-free detection. Researchers have explored various two-dimensional materials such as graphene,^21−24^ silicene,^25,26^ phosphorene,^27^ h-BN,^28,29^ Ti_2_CO_2_ MXene,^30^ Ti_3_C_2_ MXene,^31^ germanene,^32^ monolayer C_2_N,^33,34^ and monolayer GaS^35^ as a sensing platforms for DNA nucleobase detection. Recently, two-dimensional transition metal dichalcogenides (TMDs) have garnered increasing attention due to their large surface-to-volume ratio and exceptional optical and electrical properties. Researchers have investigated the sensing performance of monolayer MoS_2_ for DNA detection, both experimentally and theoretically. Farimani et al.^36^ employed atomistic and quantum simulations and found that single-layer MoS_2_ (nanopore and nanochannel) exhibits extraordinary characteristics for DNA sequencing. The MoS_2_ nanopore demonstrates distinct current signals for the detection of individual nucleobases with low noise. Furthermore, single-layer MoS_2_ exhibits characteristic response in density of states and band structure when nucleobases are placed on pristine MoS_2_ and armchair MoS_2_ nanoribbons.^36^ Jin et al.^37^ reported a novel Au-modified monolayer MoS_2_ sensor that enables rapid, sensitive, and selective detection of DNA molecules. The interaction between Au and SH groups enhances DNA adsorption on MoS_2_ by approximately 1 order of magnitude.^37^ Graf et al.^38^ demonstrated the technical feasibility of fabricating freestanding MoS_2_ nanoribbons with nanopore. DNA molecules are sensed through correlated signals from the ionic current passing through the nanopore and the transverse current passing through the nanoribbon.^38^ Since nanopore sequencing technology is widely used for DNA detection; however, it suffers from many limitations. Therefore, in the present study, we tend to probe a more robust technique and material that could be cheaper and able to commercialize for this sensing activity. For this, we are trying to study on top sensor mechanism instead of nanogap and nanopore mechanism.^39,40^

The Janus monolayer MoSSe is synthesized by replacing the top layer sulfur (S) in MoS_2_ with selenium (Se) atoms.^41,43^ Transitioning from monolayer MoS_2_ to monolayer MoSSe, which breaks the out-of-plane mirror symmetry, results in a substantial vertical dipole moment. As the sensing process on two-dimensional materials primarily occurs at the surface, the presence of a dipole moment in experimentally synthesized Janus monolayer MoSSe is expected to influence the sensing performance compared with monolayer MoS_2_ in detecting DNA nucleobases. Consequently, this study aims to investigate the potential application of two-dimensional materials, primarily MoS_2_ and MoSSe monolayers,^42−44^ for the detection of hachimoji DNA bases proposed for the expansion of genetic information system^45^ using density functional theory aided with van der Waals dispersion correction.

This investigation aims to identify the optimal adsorption sites and distances for DNA nucleobases. Commonly employed theoretical parameters, including the adsorption energy and charge transfer, have been leveraged to assess the sensing capabilities. The intermolecular interactions between the MoS_2_/MoSSe layer and DNA base pairs will be scrutinized through NCI plot analysis. Before and after nucleobase adsorption, the current–voltage (*I*–*V*) characteristics will be computed using the nonequilibrium Green’s function formalism. The outcomes of this research offer valuable insights into the influence of dipole moments on the monolayer surface in the context of DNA sensing. This understanding is poised to facilitate the identification of promising candidates for DNA sensing applications.

## 2 Computational Details

First-principles calculations are conducted using density functional theory as implemented in the Vienna Ab initio Simulation Package.^46−48^ The exchange-correlation potential is treated in the generalized gradient approximation of Perdew–Burke–Ernzerhof (PBE). Projector-augmented wave potentials are utilized, considering the valence state as follows: 1s^1^ for H, 2s^2^2p^2^ for C, 2s^2^2p^3^ for N, 2s^2^2p^4^ for O, 3s^2^3p^4^ for S, 4s^2^4p^4^ for Se, and 4p^6^4d^5^5s^1^ for Mo.^49^ The van der Waals interaction is accounted for using the DFT-D3 method.^50^ The electronic wave functions are expanded in a plane-wave basis set with a cutoff energy of 500 eV. Brillouin zone integrations are performed on 3 × 3 × 1 Monkhorst–Pack *k*-meshes.^51^ All structures are relaxed, using the conjugate gradient algorithm, until the total energy and atomic forces converge to below 10^–6^ eV and 5 × 10^–3^ eV/Å, respectively.

To construct the 2D material (slab model), a vacuum layer with a thickness of 16 Å is added in the out-of-plane direction. For adsorption studies, the supercell of size 5 × 5 × 1 is employed. The adsorption strength of a base molecule on monolayer (MoS_2_/MoSSe) is evaluated in terms of the adsorption energy (*E*_ad_) of eq 1,
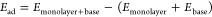
1where *E*_monolayer+base_, *E*_monolayer_, and *E*_base_ are the total energies of the combined system, monolayer (MoS_2_/MoSSe), and base molecule, respectively. The adsorption energy *E*_ad_ provides information about the stability of the adsorption between a monolayer and a base molecule. A negative *E*_ad_ value indicates an exothermic adsorption process, resulting in a favorable base/monolayer structure. For the calculations of the current, we utilize the nonequilibrium Green’s function method as implemented in the TranSIESTA method.^52^ This method employs the Landauer–Büttiker formula to calculate current, as in eq 2
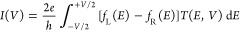
2where *f*_L_(*E*) and *f*_R_(*E*) are the Fermi distribution functions of the left and right leads, respectively, and *T*(*E*,*V*) is the transmission coefficient at energy *E* and bias voltage *V*. The electronic wave functions are expanded in a polarized double-ζ basis with a cutoff energy of 700 Ry (see Figure S1). The Brillouin zone is sampled on 1 × 5 × 51 and 1 × 5 × 1 Monkhorst-Pack *k*-meshes for the lead and transport calculations, respectively. Charge transfers are obtained by Bader charge analysis.^53^ The charge density difference plot of eq 3
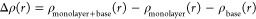
3is derived from the charge density distributions of the combined system (ρ_monolayer+base_), monolayer (ρ_monolayer_), and molecule (ρ_base_). To calculate the charge density distributions of the monolayer and molecule, we extracted the structures from the combined system.

## 3 Results and Discussion

### 3.1 Adsorption of Hachimoji DNA Bases on MoS_2_ and MoSSe Monolayers

**Figure 2:**
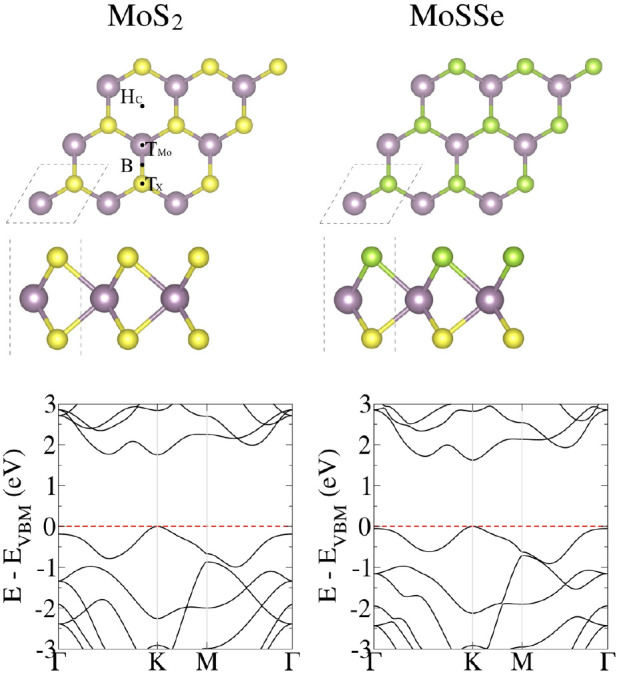
Atomic structure with electronic band structure of the hexagonal unit cell in MoS_2_ and MoSSe monolayers. The Mo, S, and Se atoms are represented by purple, yellow, and green balls, respectively.

The unit cells of the MoS_2_ and MoSSe monolayers consist of three atoms each, with Mo sandwiched between two layers of S or S and Se, as depicted in Figure 2. For the MoS_2_ monolayer, we achieved an optimized lattice constant of *a* = *b* = 3.16 Å, with average Mo–S bond lengths of 2.40 Å. The thickness of MoS_2_ is measured as 3.13 Å, and the S–Mo–S angle is determined to be 82.25^◦^. The electronic band structure exhibits characteristics of a semiconductor with a direct band gap of 1.75 eV, consistent with previous findings.^54^ For the MoSSe monolayer, the optimized lattice constants are *a* = *b* = 3.22 Å, and the Mo–S and Mo–Se bond lengths are 2.41 and 2.53 Å, respectively. The thickness of MoSSe is calculated as 3.24 Å and the dipole moment is 0.24 D in good agreement with previous reports.^55^ The electronic band structure reveals a semiconducting behavior with a direct band gap of 1.63 eV. The more diffuse orbitals of Se as compared to S atom lead to weaker overlap with the Mo orbitals, which can result in a reduction in the band gap.

In order to examine the interaction between natural DNA bases (A–C) and their modified counterparts (B–Z) on MoS_2_ and MoSSe monolayers, our initial step involves optimizing the molecular structures of the bases. The centers of mass of these molecules are kept at various possible adsorption sites on MoS_2_ and MoSSe monolayers with different orientations, and the lowest-energy configurations are obtained. All possible adsorption sites (H_C_: hexagonal center, B: bridge, T_Mo_: top of Mo atom, T_X_: top of S or Se atom) are shown in Figure 2.

**Figure 3:**
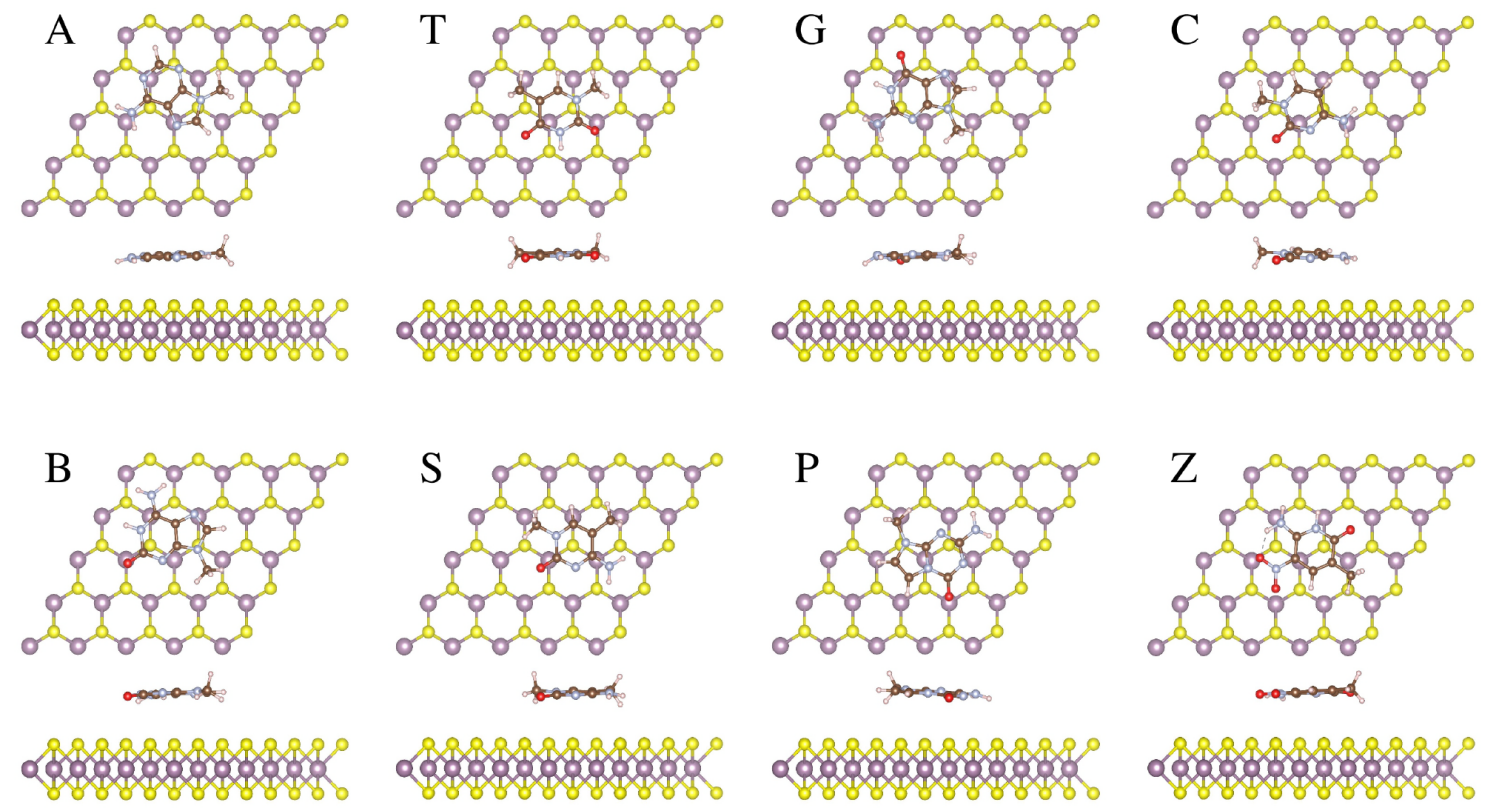
Lowest-energy configurations of natural (A, T, G, C) and modified (B, S, P, Z) base molecules on the MoS_2_ monolayer, with both top and side views. Mo, S, C, N, O, and H atoms are represented by purple, yellow, brown, gray, red, and pink balls, respectively.

The lowest-energy configurations of the natural and modified base molecules on the MoS_2_ monolayer are shown in Figure 3. All bases prefer a horizontal orientation with a binding distance of ≈3.0 Å from the monolayer surface. Additionally, we included the adsorption energy values for the various binding sites in Table S1.

In the MoS_2_ monolayer, A, T, and G bases prefer H_C_, B, and H_C_ sites, respectively, with adsorption energies of −0.789 eV, −0.748 eV, and −0.953 eV. The C base favors the T_Mo_ site with an adsorption energy of −0.756 eV, while the modified B base prefers the H_C_ site with an adsorption energy of −0.930 eV. Base S adsorbs on the top of the S atom site (T_S_) with an adsorption energy of −0.851 eV. The modified base P molecule achieves its lowest energy at the T_Mo_ site with an adsorption energy of −0.912 eV, and the base Z molecule at the B site with an adsorption energy of −0.876 eV. Notably, bond lengths in MoS_2_ and base molecules show no modification except for the N–H bond length in G and B bases, indicating physisorption.

The adsorption energy results, summarized in Table 1, indicate that all of the bases primarily exhibit physisorption. Notably, among the natural and modified bases studied, base G and base T exhibit the highest and lowest affinities for adsorption, respectively, with the following trend: G > B > P > Z > S > A > C > T. It is worth highlighting that the modified bases considered in this investigation display strong adsorption on all monolayers, as illustrated in Table 1. The adsorption energy trend for natural bases (G > A > C > T) aligns closely with findings from prior studies.^56^ This phenomenon can be attributed to the relatively larger size of purine bases in comparison to pyrimidines, leading to an increased surface area for interaction with the monolayer and consequently resulting in higher adsorption energies, with the exception of base A. Furthermore, the observed trends in adsorption energies exhibit a direct correlation with the number of heteroatoms within the base molecules.

**Table 1:** Preferred Adsorption Site, Binding Distance (*D*_h_) (Shortest Atom-to-Atom Distance Between Monolayer and Base Molecule), Adsorption Energy (*E*_ad_), and Charge Transfer (Δ*Q*: ∓ Sign Indicates Charge Depletion from or Charge Accumulation on Base Molecule) of the Hachimoji Bases Adsorbed on the MoS_2_/MoSSe Monolayer

	MoS_2_				MoSSe_S				MoSSe_Se			
base	site	*D*_h_ (Å)	*E*_ad_ (eV)	Δ*Q* (e)	site	*D*_h_ (Å)	*E*_ad_ (eV)	Δ*Q* (e)	site	*D*_h_ (Å)	*E*_ad_ (eV)	Δ*Q*(e)
A	H_C_	3.00	–0.789	–0.011	H_C_	2.99	–0.780	–0.003	H_C_	3.13	–0.804	0.006
T	B	2.88	–0.748	0.012	B	2.88	–0.742	0.017	B	2.97	–0.771	0.030
G	H_C_	2.88	–0.953	–0.007	H_C_	2.83	–0.951	0.003	H_C_	3.04	–0.974	0.016
C	T_Mo_	2.89	–0.756	–0.012	T_Mo_	3.01	–0.744	–0.004	T_Mo_	3.02	–0.779	0.004
B	H_C_	3.10	–0.930	–0.004	H_C_	2.89	–0.904	0.006	H_C_	3.09	–0.944	0.019
S	T_S_	2.78	–0.851	–0.007	B	2.94	–0.825	–0.001	T_Se_	2.98	–0.861	0.013
P	T_Mo_	2.94	–0.912	–0.011	T_Mo_	3.20	–0.896	–0.006	T_Mo_	3.35	–0.928	0.006
Z	B	3.01	–0.876	0.016	B	3.02	–0.869	0.023	B	3.20	–0.894	0.037

**Figure 4:**
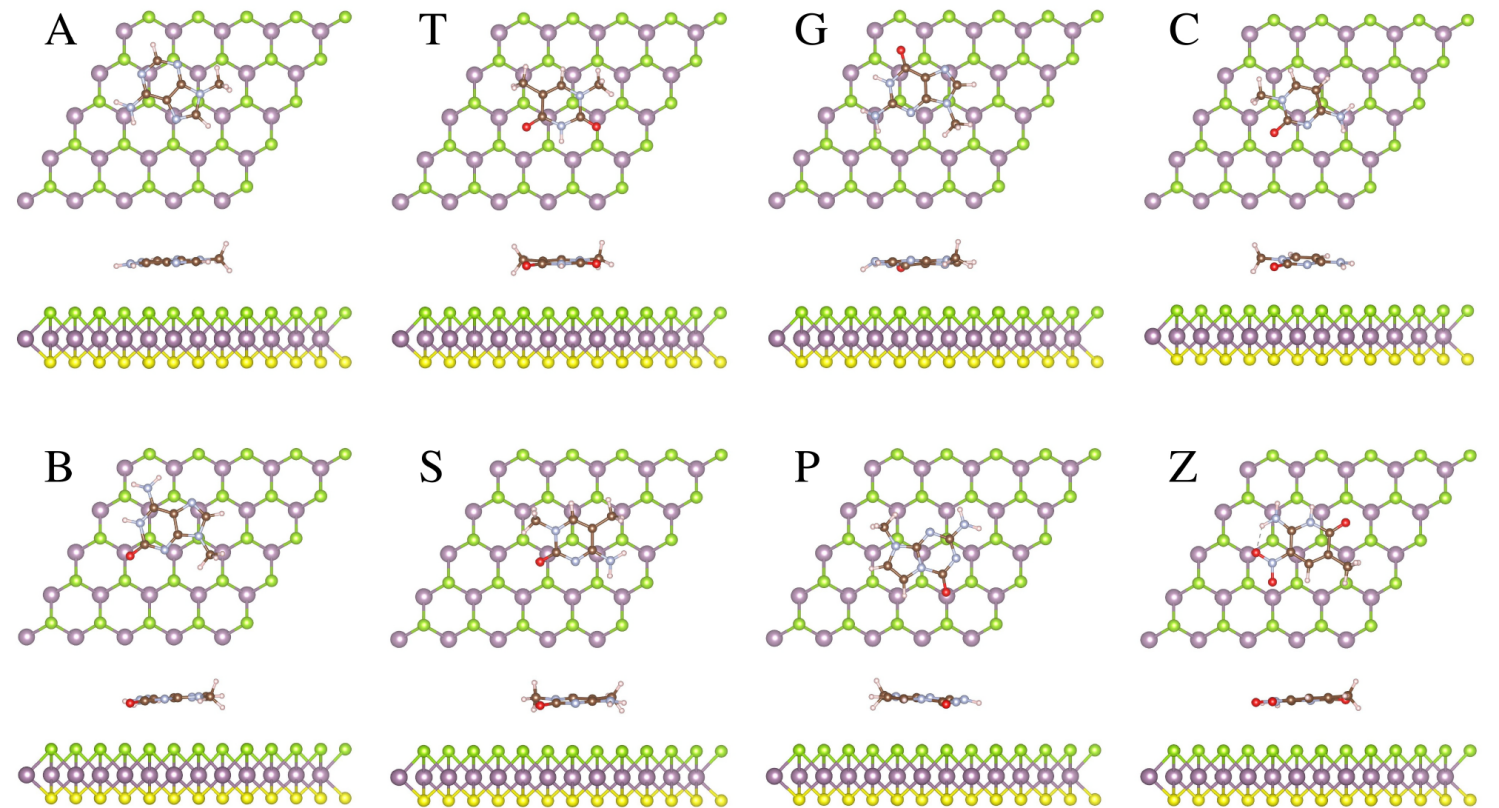
Top and side perspectives of the lowest-energy conformations of natural (A, T, G, and C) and modified (B, S, P, and Z) base molecules on the selenium (Se) side of the MoSSe monolayer. Mo, S, Se, C, N, O, and H atoms are depicted by purple, yellow, green, brown, gray, red, and pink balls, respectively.

In the context of monolayer MoSSe, we examined the adsorption behavior of natural and modified bases on both sides, denoted as S (MoSSe_S) and Se (MoSSe_Se). As illustrated in Figure S2, adsorption attributes were analyzed for all DNA bases on the S side of the MoSSe monolayer. These investigations unveiled trends and behaviors that closely mirrored those observed in the context of the monolayer MoS_2_, and thus, we do not discuss them further. Differently, unique behavior was discerned by analyzing adsorption on the Se side (see Figure 4). As illustrated in Table 1, akin to the S side of both MoS_2_ and MoSSe, the Se side of the MoSSe monolayer displayed a noticeably higher attraction for the DNA base G. All of the hachimoji bases exhibit robust interactions with the Se side of the MoSSe monolayer. Noteworthy, the adsorption energy on the Se side of MoSSe is approximately 3% higher (or lower for the S side) in comparison to the MoS_2_ monolayer. Crucially, the same consistent adsorption energy trends (G > B > P > Z > S > A > C > T) are observed for both MoS_2_ and MoSSe monolayers, irrespective of the side considered (S or Se).

**Figure 5:**
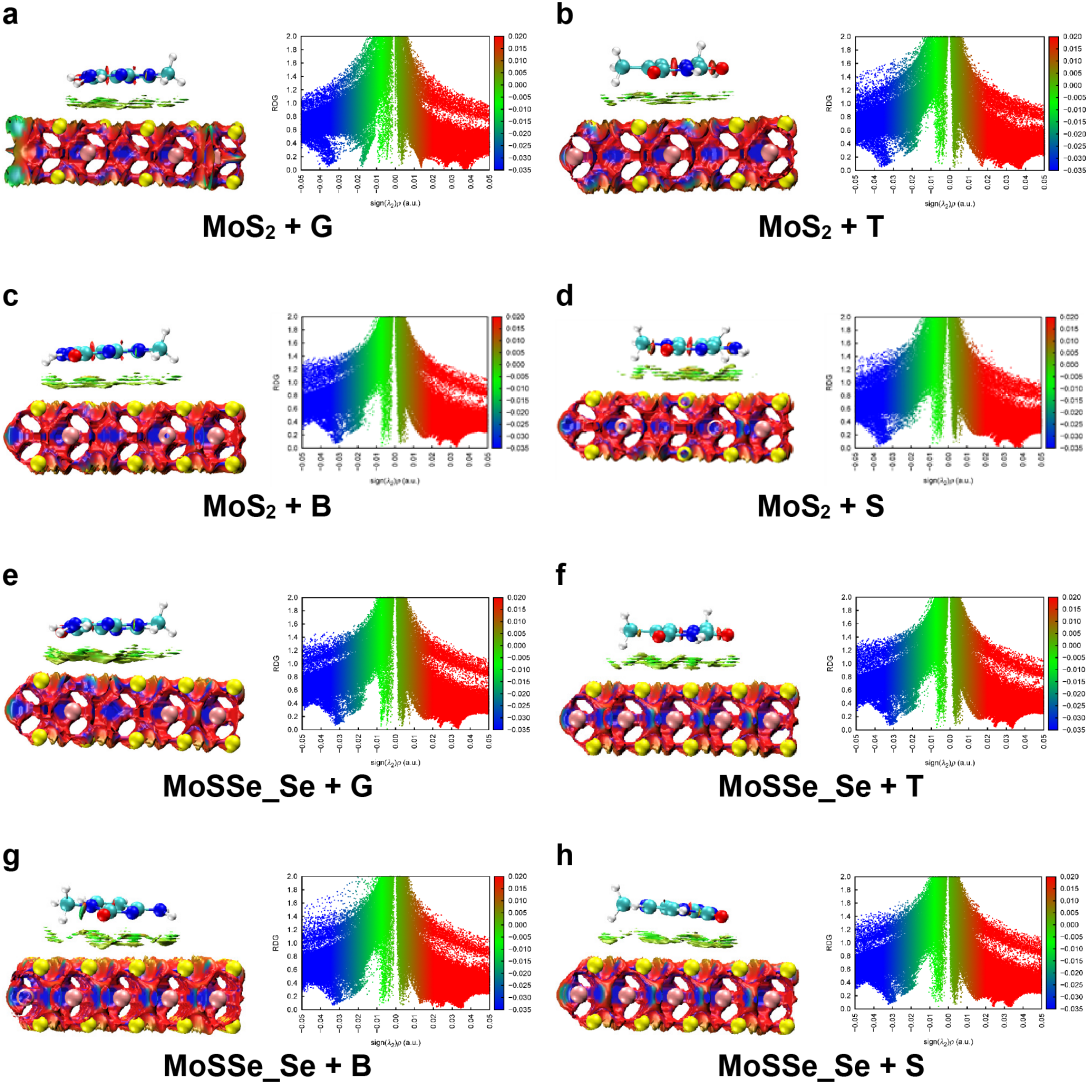
Visualization of 3D-NCI plots depicting interaction profiles for (a) G base, (b) T base, (c) B base, and (d) S base on the MoS_2_ surface. Additionally, (e) G base and (f) T base, along with (g) B base and (h) S base on MoSSe_Se surface. The accompanying neighboring graph showcases 2D-NCI plots representing the reduced density gradient (s) against the sign of the Laplacian of electron density (l2*r) in atomic units.

To differentiate between distinct modes of interaction between nucleobases and surfaces, we employ non-covalent interaction (NCI) plots, as originally introduced by Contreras-García et al.^57^ and computational details is given in the Supporting Information. These plots are presented in Figure 5 for molecules exhibiting varying degrees of adsorption. The NCI plot for the remaining base pairs is displayed in Figure S3. The NCI plot clearly illustrates the presence of weak van der Waals interactions between the base pairs and surfaces with no chemical bonds observed between them.

### 3.2 Charge Redistribution in Hachimoji DNA Base Adsorption on MoS_2_ and MoSse Monolayers

**Figure 6:**
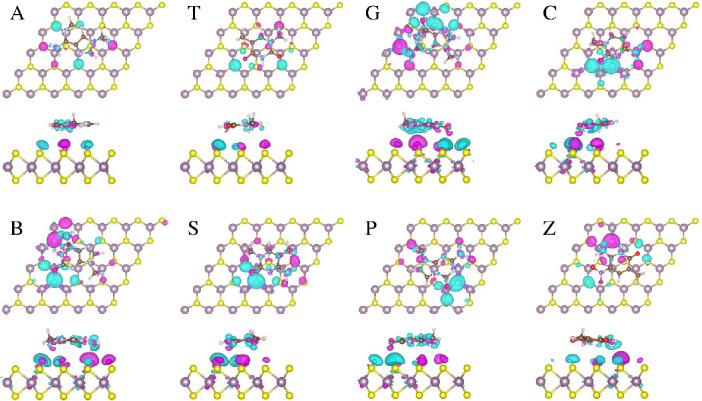
Top and side views of the charge density difference induced by adsorption base molecules on the MoS_2_ monolayer. Magenta and cyan isosurfaces represent charge accumulation and depletion, respectively (isosurface value: 5.0 × 10^–4^ electrons/Å^3^).

**Figure 7:**
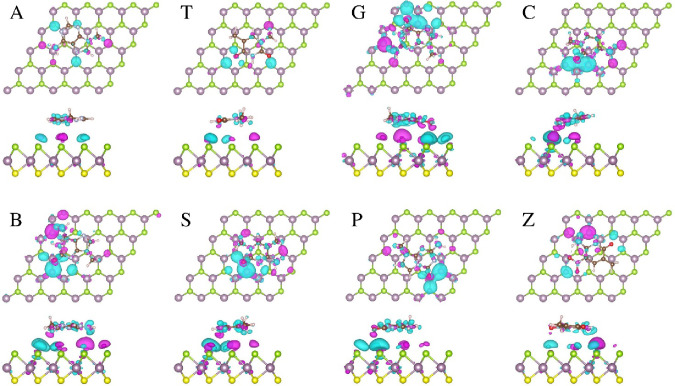
Top and side views of the charge density difference induced by adsorption base molecules on the MoSSe_Se monolayer. Magenta and cyan isosurfaces represent charge accumulation and depletion, respectively (isosurface value: 5.0 × 10^–4^ electrons/Å^3^).

To gain a deeper understanding of the interaction’s nature, we have depicted charge density difference plots in Figures 6 and 7, resulting from the adsorption of the bases on both monolayer MoS_2_ and MoSSe (Se side). These visual representations effectively illustrate how the adsorption of the bases induces a redistribution of charge density, leading to a substantial overlap of electron clouds between the base molecule and the monolayers. Notably, this charge density accumulation aligns closely with the adsorption energy trends for both monolayers, with the exception of base C. The extent of charge transfer between the bases and the MoS_2_/MoSSe monolayer is quantified using Bader charge analysis, and the corresponding values are summarized in Table 1. This variation in charge redistributions, observed for different base molecules after adsorption on MoS_2_ (Figure 6) and MoSSe_Se (Figure 7) monolayers, is anticipated to exert a significant influence on the current–voltage characteristics. These insights hold promise for facilitating the identification of specific bases for practical applications.

**Figure 8:**
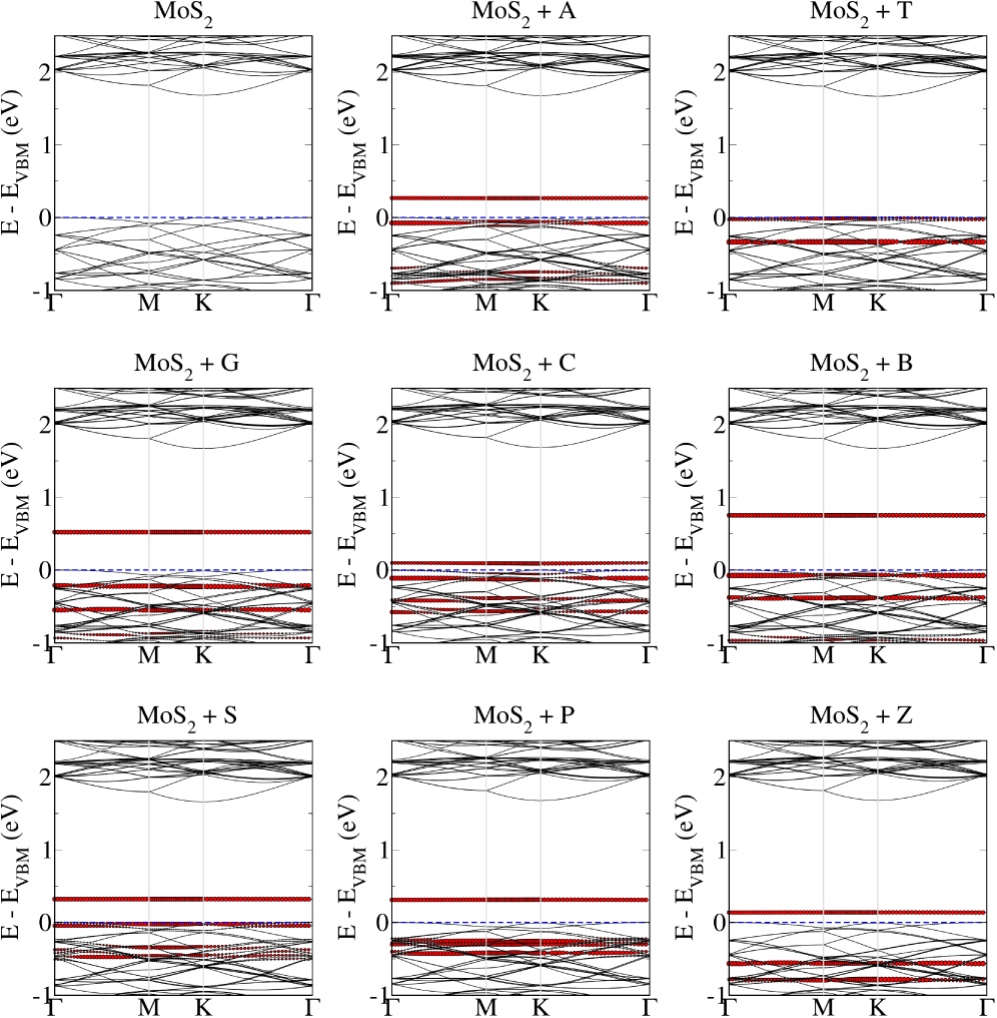
Electronic band structure of the natural (A, T, G, C) and modified (B, S, P, Z) base molecules on the MoS_2_ monolayer. Molecular contributions are highlighted in red color.

**Figure 9:**
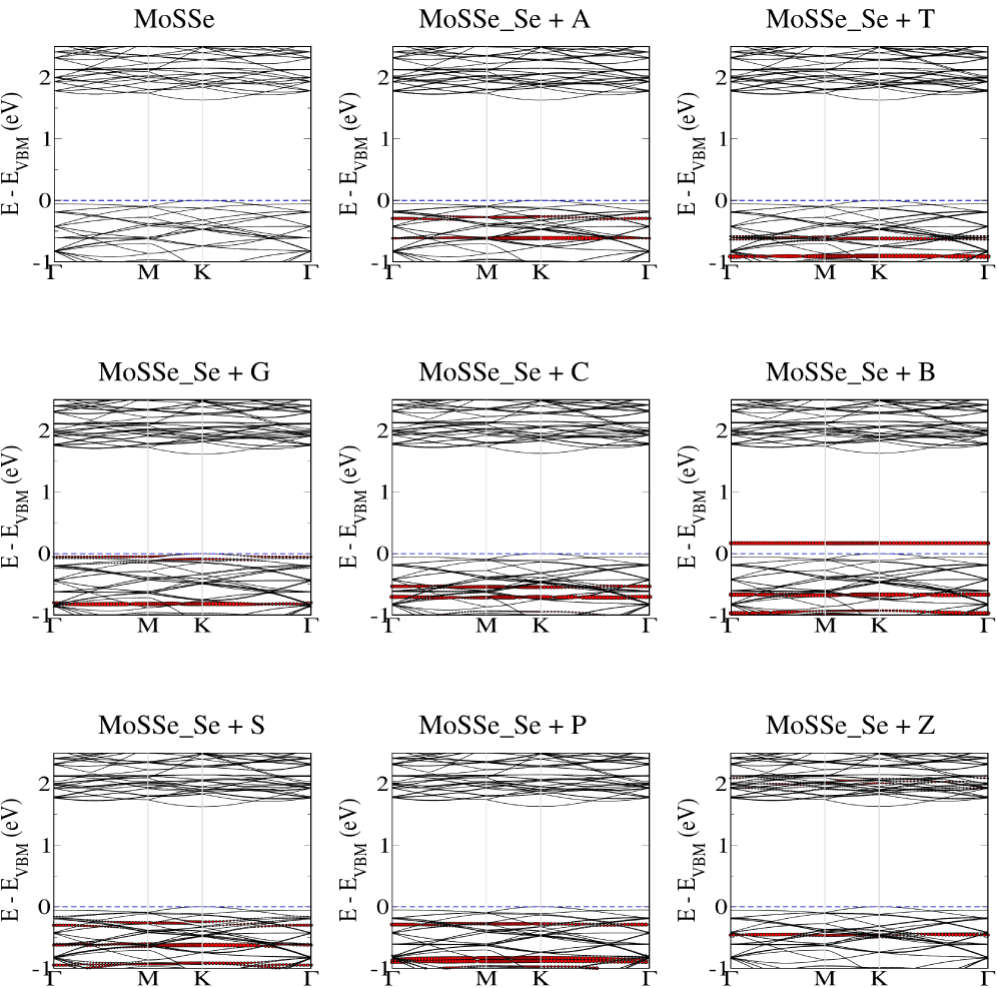
Electronic band structure of the natural (A, T, G, C) and modified (B, S, P, Z) base molecules on the MoSSe_Se monolayer. Molecular contributions are highlighted in red color.

Figure 8 illustrates the modification in the electronic band structure of monolayer MoS_2_ after bases adsorption. We note that after adsorption molecular states are being introduced in between the band gap of pristine MoS_2_, which further lowers the band gap and hence will influence the electronic transport behavior. Due to similar adsorption behavior of the bases on the S side of MoSSe monolayer, similar electronic band structure features are observed (see Figure S4). Interestingly, in the case of monolayer MoSSe, no molecular states are introduced in between the band gap, except for base B (see Figure 9). These electronic band structure features show that the electronic transport behavior for monolayer MoS_2_ should be similar to S side of MoSSe, while for Se side of monolayer MoSSe, we expect to observe different behavior. Therefore, for further electronic transport studies, we only considered monolayer MoS_2_ and the Se side of monolayer MoSSe.

### 3.3 Sensing Potential of MoS_2_/MoSSe Monolayers for Hachimoji DNA Bases

**Figure 10:**
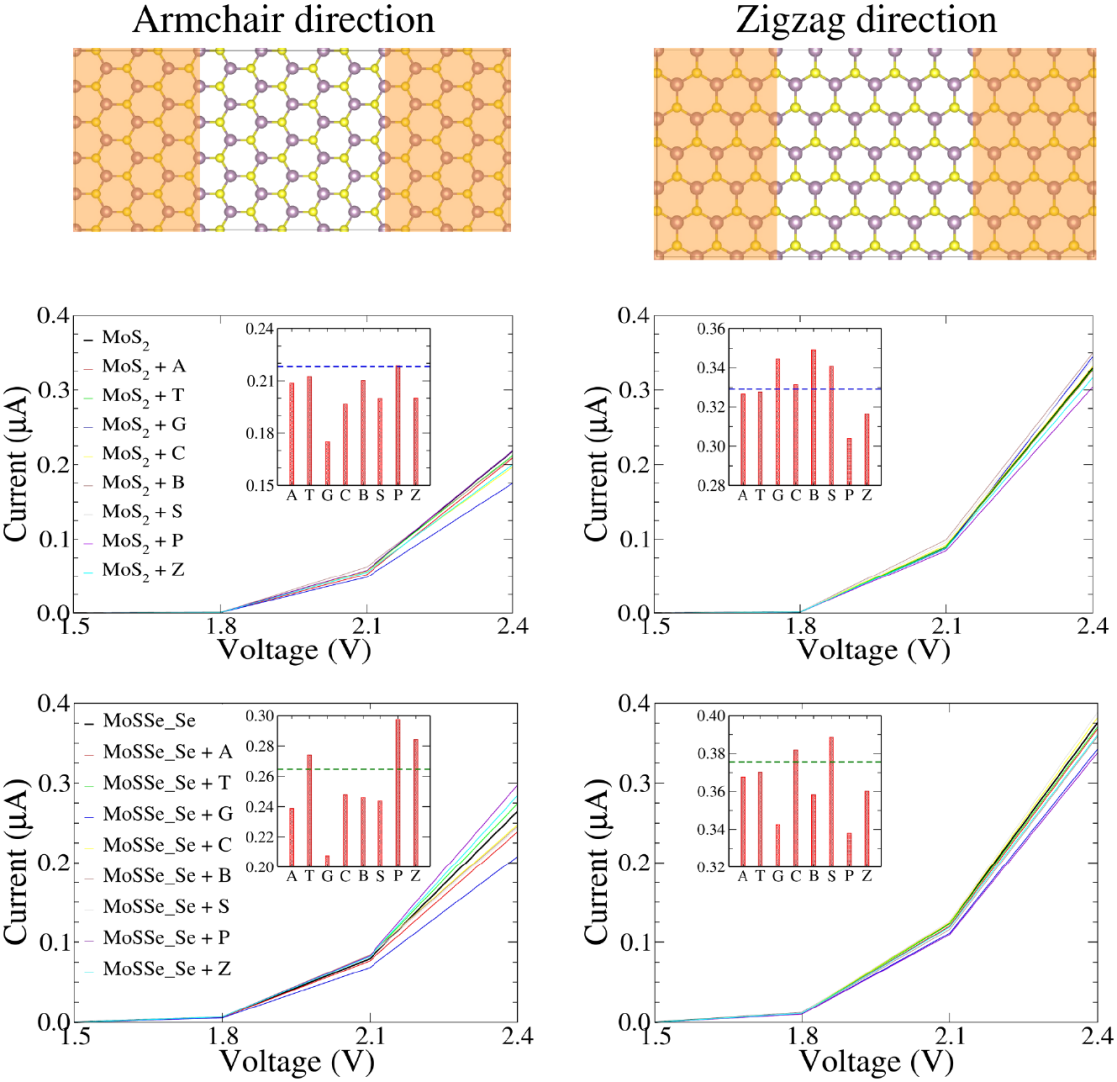
Transport setup and current–voltage characteristics of MoS_2_ and MoSSe_Se monolayers in armchair and zigzag directions, with and without adsorbed base molecules. The insets display the current value for base molecules on both monolayers at an applied voltage of 2.4 V. The horizontal line represents the current value for pure monolayers.

Quantitative assessment of the sensing potential of biosensor made up of MoS_2_/MoSSe monolayer for base molecule detection can be achieved by analyzing the changes in current–voltage characteristics (resistivity change) before and after the adsorption of base molecules. In our study, we consider the flow of current in both the armchair and zigzag directions using a setup comprising semi-infinite left and right leads connected to a central scattering region (Figure 10). For monolayer MoS_2_, the dimensions of the leads are 15.82 Å × 10.96 Å (armchair) and 16.44 Å × 9.49 Å (zigzag), and the scattering region have sizes of 15.82 Å × 16.44 Å (armchair) and 16.44 Å × 15.82 Å (zigzag), respectively. In case of monolayer MoSSe (Se side), the dimensions of the leads are 16.14 Å × 11.18 Å (16.78 Å × 9.70 Å) and the scattering region have a size of 16.14 Å Å × 16.78 Å (16.78 Å × 16.14 Å) for armchair (zigzag) direction. For both the monolayers, the current behavior is obtained at bias ranging from 0.0 to 2.4 V, with a step size of 0.3 V bias. The transport setup and resulting current–voltage characteristics, without and with adsorbed base molecules for both armchair and zigzag directions, are shown in Figure 10.

Both monolayers exhibit anisotropic electronic transport behavior, with higher current observed along the zigzag direction in contrast to the armchair direction. This intriguing phenomenon can be attributed to the substantial transmission coefficient favoring the zigzag direction, which, in turn, provides a more conductive channel for electron transport compared to the armchair direction. Furthermore, the pronounced dispersion in the electronic band along the zigzag direction suggests a lower effective mass, thus resulting in a higher current in comparison to the armchair direction (as illustrated in Figure S5). The transmission coefficient plots at applied zero bias voltage, following the adsorption of bases on both monolayers, are depicted in Figure S6. These plots reveal that within the selected energy range for the studied monolayers, different bases exhibit distinctive transmission profiles, indicative of varying currents (as defined in eq 2). This divergence underscores their potential for selective base detection.

In the context of electronic transport, the band gap plays a critical role in determining the current flow in monolayer MoSSe (Se side) in comparison to the MoS_2_ monolayer, as depicted in Figure 10. It is important to note that current flow cannot occur when the applied bias voltage is within the range of the band gap. Up to an applied bias of 1.5 V, no significant current is observed in both monolayers. At 1.8 V, the MoS_2_ monolayer exhibits negligible current flow, while in the case of the MoSSe_Se monolayer, a current on the order of 0.001 μA is detected, consistent with their respective band gap values. The narrower band gap of monolayer MoSSe results in an earlier onset of current compared to MoS_2_. With further increases in bias voltage, both monolayers exhibit a significant increase in current, with MoSSe monolayer demonstrating higher current levels.

At an applied voltage of 2.4 V along the armchair direction in the MoS_2_ monolayer, the maximum (minimum) current is observed for the P (G) base, while along the zigzag direction, the maximum (minimum) current corresponds to the B (P) base. The order of currents for the studied bases along the armchair direction increases as follows: P > T > B > A > Z > S > C > G. Along the zigzag direction, the order is B > G > S > C > T > A > Z > P. Conversely, in the case of the MoSSe_Se monolayer at 2.4 V, the maximum current along the armchair (zigzag) direction is obtained for the P (S) base, while the minimum current corresponds to the G (P) base. Along the armchair direction, the order of current is P > Z > T > C > B > S > A > G. Along the zigzag direction, the trend is S > C > T > A > Z > B > G > P.

The strong adsorption of the G base to both monolayers results in increased resistance to electron flow, consequently reducing the current following adsorption on the sensing platform compared with the pristine monolayers. At 2.4 V, the adsorption of base G on MoS_2_ (Se side of MoSSe) decreases the current along the armchair direction by 20% (21.50%) and increases (reduces) it along the zigzag direction by 5% (9%), respectively. The adsorption of base T on MoS_2_ (Se side of MoSSe) results in a 3% reduction (4% increase) in current along the armchair direction and a 0.5% reduction (1.5% reduction) in current along the zigzag direction, respectively. These variations highlight the impact of base adsorption on the electronic transport characteristics, demonstrating the potential for the selective base detection.

To create an effective DNA base sensor, the primary requirement is the ability to detect a specific base. The sensitivity of a given monolayer to a particular base molecule is quantified by assessing the change in current relative to the baseline current (corresponding to the pristine monolayer). This assessment is achieved through the relationship of eq 4:
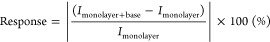
4where *I*_monolayer+base_ represents the current flow in the monolayer upon adsorption of a particular base and *I*_monolayer_ represents the current flow in the pristine monolayer. The resulting response values for base molecules, subsequent to adsorption onto the biosensors composed of both MoS_2_ and MoSSe (Se side) as the sensing platform at an applied voltage of 2.4 V.

**Figure 11:**
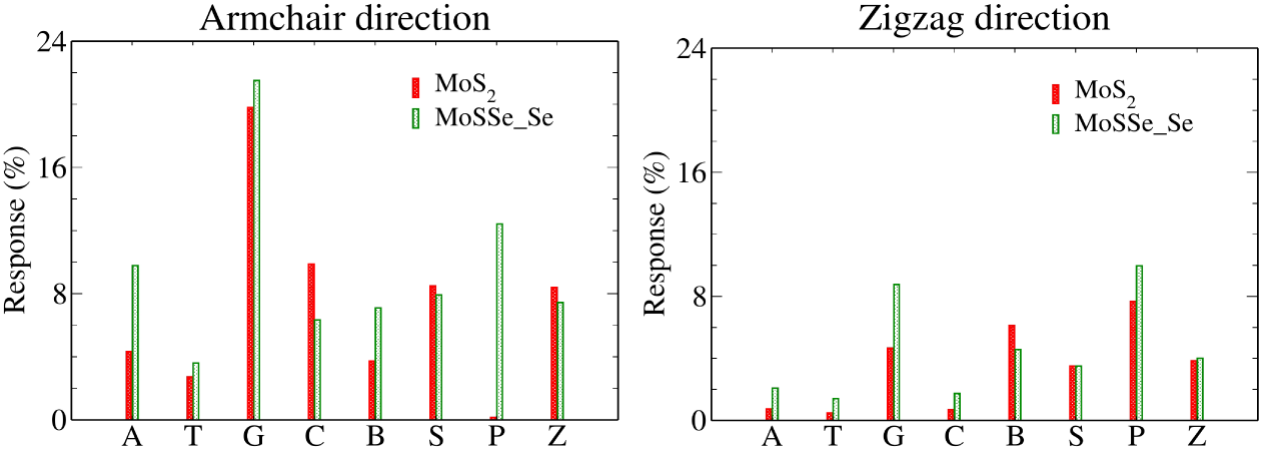
Sensitivity plots for base molecules on monolayers of MoS_2_ and MoSSe (Se side) at an applied voltage of 2.4 V.

Intriguingly, while the current values are higher along the zigzag direction for both MoS_2_ and MoSSe monolayers, the armchair direction exhibits a more pronounced response in terms of selectivity. Specifically, at an applied voltage of 2.4 V, the G base demonstrates the highest response on both monolayers, whereas the T base exhibits the lowest response post adsorption. Figure 11 illustrates that along the armchair direction, the MoSSe monolayer (Se side) effectively discriminates between natural bases with maximum and minimum current responses for bases G and T, respectively. Moreover, it distinguishes hachimoji bases (B, P, S, Z) from natural bases (A, T, G, C), though only base P is selectively identified, while bases B, S, and Z are indistinguishable. Conversely, along the zigzag direction, the MoSSe monolayer identifies only base G, with A, T, and C being indistinguishable. Among hachimoji bases, bases B and P are distinguishable, while bases S and Z are indistinguishable. For the MoS_2_ monolayer, along the armchair direction, natural bases can be separated from each other and are distinguishable from hachimoji bases except for base P, whose response falls below 1%. However, along the zigzag direction of the MoS_2_ monolayer, only bases G, B, and P are distinguishable, while bases S and Z are indistinguishable. Notably, the low response percentage (<1%) makes it challenging to detect bases A, T, and C.

Comparing response plots between MoS_2_ and MoSSe (Se side) based biosensors, it is evident that, in both directions, the MoSSe monolayer outperforms the MoS_2_ monolayer, except in cases involving base C along the armchair direction and base B along the zigzag direction.

For the comparative analysis, we compared our results with a recent study on the detection of Hachimoji nucleobases using a hybrid graphene/h-BN nanopore device.^58^ The previous study reported transconductance sensitivity values, whereas we calculated the current sensitivity values in our study. We presented the transconductance sensitivity value for our materials using a gate voltage of +1.38 V (+1.20 V) for MoS_2_ (MoSSe_Se). Hybrid graphene/h-BN exhibited superior sensitivity compared to both MoS_2_ and MoSSe_Se, with B nucleobase demonstrating the highest sensitivity, followed by S, P, and Z nucleobases.^58^ However, when comparing the adsorption energy values, we observed weak interactions of Hachimoji bases with the graphene/h-BN nanopore, with a positive binding energy of +0.05 eV detected for base P. Base Z and ribose S (rS) exhibited binding energies of −0.02 eV, which are lower than the thermal energy at room temperature. These findings suggest that some bases, such as P, Z, and rS, may be challenging to detect using the hybrid graphene/h-BN nanopore, while B and S can be selectively detected. Conversely, these bases showed moderate interaction with the MoS_2_ (MoSSe_Se) materials presented in this work.

## 4 Conclusions

In this study, we have employed density functional theory and nonequilibrium Green’s function to explore the potential for natural and modified DNA nucleobase sensing on monolayers of MoS_2_ and MoSSe. Our analysis reveals that both MoS_2_ and MoSSe monolayers exhibit moderate adsorption energies for a range of nucleobases, positioning them as promising candidates for DNA sequencing applications. Of particular note is the strong adsorption affinity of base G on both monolayers. These findings collectively indicate that MoSSe (Se side) monolayers hold promise as a superior platform for the selective detection of DNA bases. The strong adsorption of certain bases impacts the electron flow, and the observed variations in current can be harnessed for practical applications.

In summary, our research contributes to the development of efficient hachimoji base sensors, providing a foundation for the design of novel biosensing devices with improved selectivity and sensitivity. The insights gained from this study can be instrumental in advancing the field of personalized medicine, where precise DNA analysis is essential for tailoring medical treatments and therapies.

## Supporting Information Available

Computational details for NCI plots, adsorption energy values for different binding sites, top and side views of the lowest-energy conformations of natural (A, T, G, C) and modified (B, S, P, Z) base molecules on the S side of the MoSSe monolayer, NCI surface and NCI plot illustrating the base monolayer interactions, electronic band structure of the natural (A, T, G, C) and modified (B, S, P, Z) base molecules on S side of MoSSe monolayer, atomic structure with electronic band structure of rectangular unit cell of MoS_2_ and MoSSe monolayers, and transmission coefficient plots at applied zero bias voltage for MoS_2_ and MoSSe (Se side) along armchair and zigzag directions without and with base molecules (PDF)

## References

[ref1] Li F.; Huang Y.; Yang Q.; Zhong Z.; Li D.; Wang L.; Song S.; Fan C. A Graphene-Enhanced Molecular Beacon for Homogeneous DNA Detection. Nanoscale 2010, 2 (6), 1021–1026. 10.1039/b9nr00401g. 20648302

[ref2] He S.; Song B.; Li D.; Zhu C.; Qi W.; Wen Y.; Wang L.; Song S.; Fang H.; Fan C. A Graphene Nanoprobe for Rapid, Sensitive, and Multicolor Fluorescent DNA Analysis. Adv. Funct. Mater. 2010, 20 (3), 453–459. 10.1002/adfm.200901639.

[ref3] Kumar S. Epigenomics of Plant Responses to Environmental Stress. Epigenomes 2018, 2 (1), 6 10.3390/epigenomes2010006.

[ref4] Hikida Y.; Kimoto M.; Yokoyama S.; Hirao I. Site-Specific Fluorescent Probing of RNA Molecules by Unnatural Base-Pair Transcription for Local Structural Conformation Analysis. Nat. Protoc. 2010, 5, 1312–1323. 10.1038/nprot.2010.77. 20595959

[ref5] Kimoto M.; Mitsui T.; Yamashige R.; Sato A.; Yokoyama S.; Hirao I. A New Unnatural Base Pair System Between Fluorophore and Quencher Base Analogues for Nucleic Acid-Based Imaging Technology. J. Am. Chem. Soc. 2010, 132, 15418–15426. 10.1021/ja1072383. 20939572

[ref6] Kimoto M.; Mitsui T.; Yokoyama S.; Hirao I. A Unique Fluorescent Base Analogue for the Expansion of the Genetic Alphabet. J. Am. Chem. Soc. 2010, 132, 4988–4989. 10.1021/ja100806c. 20334374

[ref7] Hoshika S.; Chen F.; Leal N. A.; Benner S. A. Artificial Genetic Systems: Self-Avoiding DNA in PCR and Multiplexed PCR. Angew. Chem., Int. Ed. 2010, 49, 5554–5557. 10.1002/anie.201001977. PMC6027612 20586087

[ref8] Sherrill C. B.; Marshall D. J.; Moser M. J.; Larsen C. A.; Daude-Snow L.; Prudent J. R. Nucleic Acid Analysis using an Expanded Genetic Alphabet to Quench Fluorescence. J. Am. Chem. Soc. 2004, 126, 4550–4556. 10.1021/ja0315558. 15070373

[ref9] Kimoto M.; Endo M.; Mitsui T.; Okuni T.; Hirao I.; Yokoyama S. Site-Specific Incorporation of a Photo-Crosslinking Component into RNA by T7 Transcription Mediated by Unnatural Base Pairs. Chem. Biol. 2004, 11, 47–55. 10.1016/j.chembiol.2003.12.016. 15112994

[ref10] Kimoto M.; Kawai R.; Mitsui T.; Yokoyama S.; Hirao I. An Unnatural Base Pair System for Efficient PCR Amplification and Functionalization of DNA Molecules. Nucleic Acids Res. 2009, 37, e14 10.1093/nar/gkn956. 19073696 PMC2632903

[ref11] Kimoto M.; Mitsui T.; Harada Y.; Sato A.; Yokoyama S.; Hirao I. Fluorescent Probing for RNA Molecules by an Unnatural Base-Pair System. Nucleic Acids Res. 2007, 35, 5360–5369. 10.1093/nar/gkm508. 17693436 PMC2018647

[ref12] Flamme M.; Röthlisberger P.; Levi-Acobas F.; Chawla M.; Oliva R.; Cavallo L.; Gasser G.; Marlière P.; Herdewijn P.; Hollenstein M. Enzymatic Formation of an Artificial Base Pair Using a Modified Purine Nucleoside Triphosphate. ACS Chem. Biol. 2020, 15 (11), 2872–2884. 10.1021/acschembio.0c00396. 33090769

[ref13] Chawla M.; Poater A.; Besalú-Sala P.; Kalra K.; Oliva R.; Cavallo L. Theoretical Characterization of Sulfur-to-Selenium Substitution in an Emissive RNA Alphabet. Phys. Chem. Chem. Phys. 2018, 20, 7676–7685. 10.1039/C7CP07656H. 29497733

[ref14] Chawla M.; Poater A.; Oliva R.; Cavallo L. Structural and Energetic Characterization of the Emissive RNA Alphabet Based on the Isothiazolo[4,3-d]Pyrimidine Heterocycle Core. Phys. Chem. Chem. Phys. 2016, 18, 18045–18053. 10.1039/C6CP03268K. 27328414

[ref15] Chawla M.; Credendino R.; Oliva R.; Cavallo L. Structural and Energetic Impact of Non-Natural 7-Deaza-8-Azaadenine and Its 7-Substituted Derivatives on H-Bonding Potential with Uracil in RNA Molecules. J. Phys. Chem. B 2015, 119 (41), 12982–12989. 10.1021/acs.jpcb.5b06861. 26389789

[ref16] Chawla M.; Gorle S.; Shaikh A. R.; Oliva R.; Cavallo L. Replacing Thymine with a Strongly Pairing Fifth Base: A Combined Quantum Mechanics and Molecular Dynamics Study. Comput. Struct. Biotechnol. J. 2021, 19, 1312–1324. 10.1016/j.csbj.2021.02.006. 33738080 PMC7940798

[ref17] Chawla M.; Minenkov Y.; Vu K. B.; Oliva R.; Cavallo L. Structural and Energetic Impact of Non-natural 7-Deaza-8-azaguanine, 7-Deaza-8-azaisoguanine, and Their 7-Substituted Derivatives on Hydrogen-Bond Pairing with Cytosine and Isocytosine. ChemBiochem 2019, 20 (17), 2262–2270. 10.1002/cbic.201900245. 30983115

[ref18] Hoshika S.; Leal N. A.; Kim M.-J.; Kim M.-S.; Karalkar N. B.; Kim H.-J.; Bates A. M.; Watkins N. E. Jr; SantaLucia H. A.; Meyer A. J.; DasGupta S.; Piccirilli J. A.; Ellington A. D.; SantaLucia J. Jr; Georgiadis M. M.; Benner S. A. Hachimoji DNA and RNA: A Genetic System with Eight Building Blocks. Science 2019, 363 (6429), 884–887. 10.1126/science.aat0971. 30792304 PMC6413494

[ref19] Dhami K.; Malyshev D. A.; Ordoukhanian P.; Kubelka T.; Hocek M.; Romesberg F. E. Systematic Exploration of a Class of Hydrophobic Unnatural Base Pairs Yields Multiple New Candidates for the Expansion of the Genetic Alphabet. Nucleic Acids Res. 2014, 42 (16), 10235–10244. 10.1093/nar/gku715. 25122747 PMC4176363

[ref20] Yang Z.; Chen F.; Alvarado J. B.; Benner S. A. Amplification, Mutation, and Sequencing of a Six-Letter Synthetic Genetic System. J. Am. Chem. Soc. 2011, 133 (38), 15105–15112. 10.1021/ja204910n. 21842904 PMC3427765

[ref21] Dontschuk N.; Stacey A.; Tadich A.; Rietwyk K. J.; Schenk A.; Edmonds M. T.; Shimoni O.; Pakes C. I.; Prawer S.; Cervenka J. A graphene Field-Effect Transistor as a Molecule-Specific Probe of DNA Nucleobases. Nat. Commun. 2015, 6, 6563 10.1038/ncomms7563. 25800494

[ref22] Heerema S. J.; Dekker C. Graphene Nanodevices for DNA Sequencing. Nat. Nanotechnol. 2016, 11, 127–136. 10.1038/nnano.2015.307. 26839258

[ref23] Lee J.-H.; Choi Y.-K.; Kim H.-J.; Scheicher R. H.; Cho J.-H. Physisorption of DNA Nucleobases on h-BN and Graphene: vdW-Corrected DFT Calculations. J. Phys. Chem. C 2013, 117, 13435–13441. 10.1021/jp402403f.

[ref24] Vovusha H.; Sanyal B. Adsorption of Nucleobases on 2D Transition-Metal Dichalcogenidesand Graphene Sheet: A First Principles Density Functional Theory Study. RSC Adv. 2015, 5, 67427 10.1039/C5RA14664J.

[ref25] Amorim R. G.; Scheicher R. H. Silicene as a New Potential DNA Sequencing Device. Nanotechnology 2015, 26, 154002 10.1088/0957-4484/26/15/154002. 25797645

[ref26] Li Q.; Liu H.; Tian Y.; Guo J.; Chen G.; Lee J. Y. Methylation Detection and DNA Sequencing Based on Adsorption of Nucleobases on Silicene Nanoribbon. J. Phys. Chem. C 2020, 124, 10823–10831. 10.1021/acs.jpcc.0c01734.

[ref27] Cortés-Arriagada D. Phosphorene as a Template Material for Physisorption of DNA/RNA Nucleobases and Resembling of Base Pairs: A Cluster DFT Study and Comparisons with Graphene. J. Phys. Chem. C 2018, 122, 4870–4880. 10.1021/acs.jpcc.7b11268.

[ref28] Bhai S.; Ganguly B. Exploiting the Optical Sensing of Fluorophore-Tagged DNA Nucleobases on Hexagonal BN and Al-doped BN Sheets: A Computational Study. Phys. Chem. Chem. Phys. 2022, 24, 829 10.1039/D1CP04009J. 34928284

[ref29] Tyagi A.; Chu K.; Hossain M. D.; Abidi I. H.; Lin W.; Yan Y.; Zhang K.; Luo Z. Revealing the Mechanism of DNA Passing Through Graphene and Boron Nitride Nanopores. Nanoscale 2019, 11, 23438 10.1039/C9NR07651D. 31799536

[ref30] Gouveia J. D.; Novell-Leruth G.; Viñes F.; Illas F.; Gomes J. R. B. The Ti2CO2MXene as a Nucleobase 2D Sensor: A First-Principles Study. Appl. Surf. Sci. 2021, 544, 148946 10.1016/j.apsusc.2021.148946.

[ref31] Yadav P.; Cao Z.; Farimani A. B. DNA Detection with Single-Layer Ti3C2Mxene Nanopore. ACS Nano 2021, 15, 4861–4869. 10.1021/acsnano.0c09595. 33660990

[ref32] Rezapour M. R.; Biel B. DNA/RNA Sequencing using Germanene Nanoribbons via Two Dimensional Molecular Electronic Spectroscopy: an Ab Initio Study. Nanoscale 2022, 14, 5147–5153. 10.1039/D1NR07336B. 35302137

[ref33] Gu Z.; Zhao L.; Liu S.; Duan G.; Perez-Aguilar J. M.; Luo J.; Li W.; Zhou R. Orientational Binding of DNA Guided by the C2N Template. ACS Nano 2017, 11, 3198–3206. 10.1021/acsnano.7b00236. 28287704

[ref34] Mukhopadhyay T. K.; Bhattacharyya K.; Datta A. Gauging the Nanotoxicity of h2D-C2N Toward Single-Stranded DNA: an in Silico Molecular Simulation Approach. ACS Appl. Mater. Interfaces 2018, 10, 13805–13818. 10.1021/acsami.8b00494. 29611415

[ref35] Singh D.; Panda P. K.; Mishra Y. K.; Ahuja R. Van der Waals Induced Molecular Recognition of Canonical DNA Nucleobases on a 2D GaS Monolayer. Phys. Chem. Chem. Phys. 2020, 22, 6706 10.1039/C9CP06418D. 32162626

[ref36] Farimani A. B.; Min K.; Aluru N. R. DNA Base Detection using a Single-Layer MoS2. ACS Nano 2014, 8, 7914–7922. 10.1021/nn5029295. 25007098

[ref37] Jin K.; Xie L.; Tian Y.; Liu D. Au-Modified Monolayer MoS2 Sensor for DNA Detection. J. Phys. Chem. C 2016, 120, 11204–11209. 10.1021/acs.jpcc.6b01193.

[ref38] Graf M.; Lihter M.; Altus D.; Marion S.; Radenovic A. Transverse Detection of DNA Using a MoS2 Nanopore. Nano Lett. 2019, 19, 9075–9083. 10.1021/acs.nanolett.9b04180. 31710497

[ref39] Xue L.; Yamazaki H.; Ren R.; Wanunu M.; Ivanov A. P.; Edel J. B. Solid-state nanopore sensors. Nat. Rev. Mater. 2020, 5 (12), 931–951. 10.1038/s41578-020-0229-6.

[ref40] Qiu H.; Sarathy A.; Schulten K.; Leburton J.-P. Detection and mapping of DNA methylation with 2D material nanopores. npj 2D Mater. Appl. 2017, 1 (1), 3 10.1038/s41699-017-0005-7. 29399640 PMC5794036

[ref41] Wan X.; Chen E.; Yao J.; Gao M.; Miao X.; Wang S.; Gu Y.; Xiao S.; Zhan R.; Chen K.; et al. Synthesis and Characterization of Metallic Janus MoSH Monolayer. ACS Nano 2021, 15, 20319–20331. 10.1021/acsnano.1c08531. 34870978

[ref42] Lee Y.-H.; Zhang X.-Q.; Zhang W.; Chang M.-T.; Lin C.-T.; Chang K.-D.; Yu Y.-C.; Wang J. T.-W.; Chang C.-S.; Li L.-J.; et al. Synthesis of Large-Area MoS2 Atomic Layers with Chemical Vapor Deposition. Adv. Mater. 2012, 24 (17), 2320–2325. 10.1002/adma.201104798. 22467187

[ref43] Lu A.-Y.; Zhu H.; Xiao J.; Chuu C.-P.; Han Y.; Chiu M.-H.; Cheng C.-C.; Yang C.-W.; Wei K.-H.; Yang Y.; et al. Janus Monolayers of Transition Metal Dichalcogenides. Nat. Nanotechnol. 2017, 12, 744–749. 10.1038/nnano.2017.100. 28507333

[ref44] Zhang J.; Jia S.; Kholmanov I.; Dong L.; Er D.; Chen W.; Guo H.; Jin Z.; Shenoy V. B.; Shi L.; et al. Janus Monolayer Transition-Metal Dichalcogenides. ACS Nano 2017, 11, 8192–8198. 10.1021/acsnano.7b03186. 28771310

[ref45] Chawla M.; Credendino R.; Chermak E.; Oliva R.; Cavallo L. Theoretical Characterization of the H-Bonding and Stacking Potential of Two Nonstandard Nucleobases Expanding the Genetic Alphabet. J. Phys. Chem. B 2016, 120 (9), 2216–2224. 10.1021/acs.jpcb.6b00125. 26882210

[ref46] Kresse G.; Hafner J. Ab Initio Molecular Dynamics for Liquid Metals. Phys. Rev. B 1993, 47, 558–561. 10.1103/PhysRevB.47.558. 10004490

[ref47] Kresse G.; Furthmüller J. Efficient Iterative Schemes for Ab Initio Total-Energy Calculations using a Plane-Wave Basis Set. Phys. Rev. B 1996, 54, 11169–11186. 10.1103/PhysRevB.54.11169. 9984901

[ref48] Kresse G.; Joubert D. From Ultrasoft Pseudopotentials to the Projector Augmented-Wave Method. Phys. Rev. B 1999, 59, 1758–1775. 10.1103/PhysRevB.59.1758.

[ref49] Blöchl P. E. Projector Augmented Wave Method. Phys. Rev. B 1994, 50, 17953–17979. 10.1103/PhysRevB.50.17953. 9976227

[ref50] Grimme S.; Antony J.; Ehrlich S.; Krieg H. A Consistent and Accurate Ab Initio Parametrization of Density Functional Dispersion Correction (DFT-D) for the 94 Elements H-Pu. J. Chem. Phys. 2010, 132, 154104 10.1063/1.3382344. 20423165

[ref51] Monkhorst H. J.; Pack J. D. Special Points for Brillouin-Zone Integrations. Phys. Rev. B 1976, 13, 5188–5192. 10.1103/PhysRevB.13.5188.

[ref52] Brandbyge M.; Mozos J.-L.; Ordejón P.; Taylor J.; Stokbro K. Density-Functional Method for Nonequilibrium Electron Transport. Phys. Rev. B 2002, 65, 165401 10.1103/PhysRevB.65.165401.

[ref53] Henkelman G.; Arnaldsson A.; Jónsson H. A Fast and Robust Algorithm for Bader Decomposition of Charge Density. Comput. Mater. Sci. 2006, 36, 354–360. 10.1016/j.commatsci.2005.04.010.

[ref54] Le O. K.; Chihaia V.; Pham-Ho M.-P.; Son D. N. Electronic and Optical Properties of Monolayer MoS2 under the Influence of Polyethyleneimine Adsorption and Pressure. RSC Adv. 2020, 10, 4201–4210. 10.1039/C9RA09042H. 35495219 PMC9049067

[ref55] Zhao N.; Schwingenschlögl U. Dipole-Induced Ohmic Contacts between Monolayer Janus MoSSe and Bulk Metals. Npj 2D Mater. Appl. 2021, 5, 72 10.1038/s41699-021-00253-w.

[ref56] Sadeghi M.; Jahanshahi M.; Ghorbanzadeh M.; Najafpour G. Adsorption of DNA/RNA Nucleobases onto Single-Layer MoS2 and Li-Doped MoS2: A Dispersion-Corrected DFT Study. Appl. Surf. Sci. 2018, 434, 176–187. 10.1016/j.apsusc.2017.10.162.

[ref57] Contreras-García J.; Johnson E. R.; Keinan S.; Chaudret R.; Piquemal J.-P.; Beratan D. N.; Yang W. NCIPLOT: A Computational Tool for Visualizing Noncovalent Interaction Regions. J. Chem. Theory Comput. 2011, 7, 625–632. 10.1021/ct100641a. 21516178 PMC3080048

[ref58] De Souza F. A. L.; Sivaraman G.; Scheicher R. H.; Scopel W. L.; Fyta M.; Amorim R. G. Electrically Sensing Hachimoji DNA Nucleotides through a Hybrid Graphene/h-BN Nanopore. Nanoscale 2020, 12, 18289–18295. 10.1039/D0NR04363J. 32857078

