# Supplementary material for: Detecting Hachimoji DNA: An Eight-Building-Block Genetic System with MoS2 and Janus MoSSe Monolayers
Source: ACS Appl Mater Interfaces. 2024 Apr 18;16(17):21427–37. doi: 10.1021/acsami.3c18400 (PMC11071042; doi:10.1021/acsami.3c18400)
Supplement: Supplementary file 1 — am3c18400_si_001.pdf [file am3c18400_si_001.pdf]

Supporting Information

Detecting Hachimoji DNA: An Eight-Building Block Genetic System with MoS_2 and Janus MoSSe Monolayers

Vasudeo Babar[1], Sitansh Sharma[2,*], Abdul Rajjak Shaikh[2], Romina Oliva[3], Mohit Chawla[1], Luigi Cavallo[1,*]

[1]King Abdullah University of Science and Technology (KAUST), Physical Sciences and Engineering Division, Kaust Catalysis Center, Thuwal 23955-6900, Saudi Arabia.

[2]Department of Research and Innovation, STEMskills Research and Education Lab Private Limited, Faridabad 121002, Haryana. India.

[3]Department of Sciences and Technologies, University Parthenope of Naples, Centro Direzionale Isola C4, I-80143, Naples, Italy.

*Email: sitanshsharma@gmail.com, luigi.cavallo@kaust.edu.sa

Computational Methodology for NCI Plots

In order to comprehend the intermolecular associations between the surfaces of MoS_2 and MoSSe with various absorbed base molecules, we examined non-covalent interaction (NCI) plots, a methodology introduced by Contreras-García et al.[1] The structures optimized using the VASP software as described previously, underwent single-point energy calculations employing the Gaussian 16 program.[2] Our calculations utilized the PbePbe functional along with the 6-31+G(d) basis set for C, H, N, O and S atoms. Additionally, we employed the LanL2DZ ECP basis set for modeling the behavior of Mo, and Se atoms. On the other hand, NCI index analyses were executed through implementation of Multiwfn 3.3.9 software.[3] Subsequently, the NCI isosurfaces for the complexes were visualized utilizing VMD 1.9.3 software,[4] leveraging Gaussian cube files generated by Multiwfn.

Table S1: Adsorption energy data for different binding sites of nucleobases on MoS_2 and MoSSe surfaces.

**MoS_2 + Base**								
Site	A	T	G	C	B	S	P	Z
B	-0.785	**-0.748**	-0.932	-0.752	-0.917	-0.840	-0.907	**-0.876**
H_C	**-0.789**	-0.741	**-0.953**	-0.753	**-0.930**	-0.837	-0.902	-0.843
T_{Mo}	-0.787	-0.744	-0.927	**-0.756**	-0.915	-0.840	**-0.912**	-0.875
T_S	-0.781	-0.746	-0.949	-0.750	-0.918	**-0.851**	-0.903	-0.863
**MoSSe_S + Base**								
Site	SA	ST	SG	SC	SB	SS	SP	SZ
B	-0.773	**-0.742**	-0.921	-0.739	-0.898	**-0.825**	-0.890	**-0.869**
H_C	**-0.780**	-0.731	**-0.951**	-0.744	**-0.904**	-0.820	-0.883	-0.838
T_{Mo}	-0.780	-0.735	-0.916	**-0.744**	-0.904	-0.824	**-0.896**	-0.869
T_S	-0.776	-0.737	-0.946	-0.743	-0.902	-0.823	-0.856	-0.859
**MoSSe_Se + Base**								
Site	SeA	SeT	SeG	SeC	SeB	SeS	SeP	SeZ
B	-0.797	**-0.771**	-0.951	-0.778	-0.928	-0.860	-0.924	**-0.894**
H_C	**-0.804**	-0.768	**-0.974**	-0.779	**-0.944**	-0.861	-0.921	-0.864
T_{Mo}	-0.803	-0.765	-0.951	**-0.779**	-0.931	-0.858	**-0.928**	-0.893
T_{Se}	-0.791	-0.770	-0.971	-0.778	-0.940	**-0.861**	-0.887	-0.838

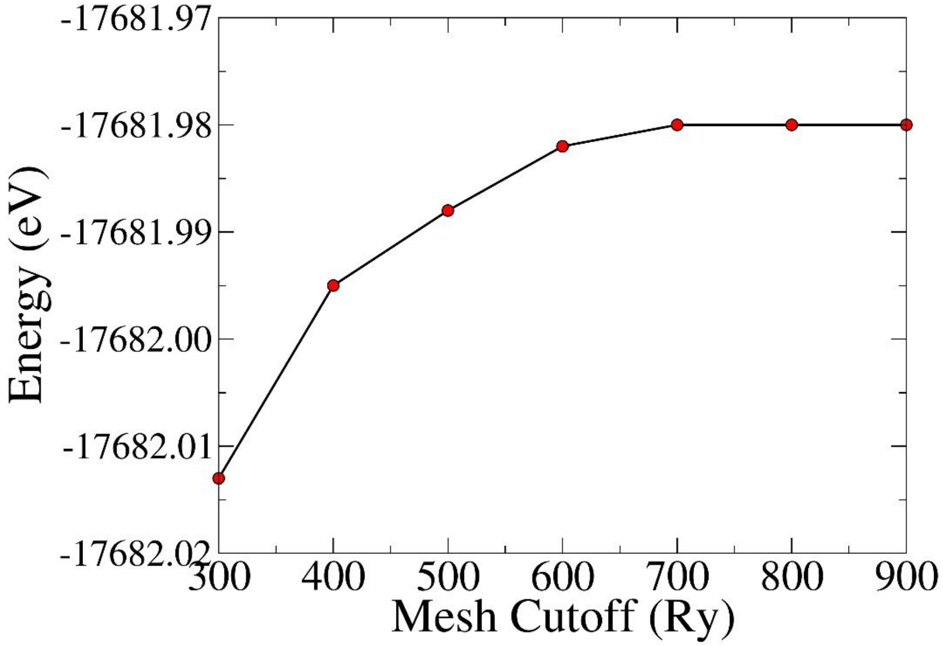

Figure S1: The plot of convergence test for energy with respect to mesh cutoff. Above graph shows energy values converged at 700 Ry.

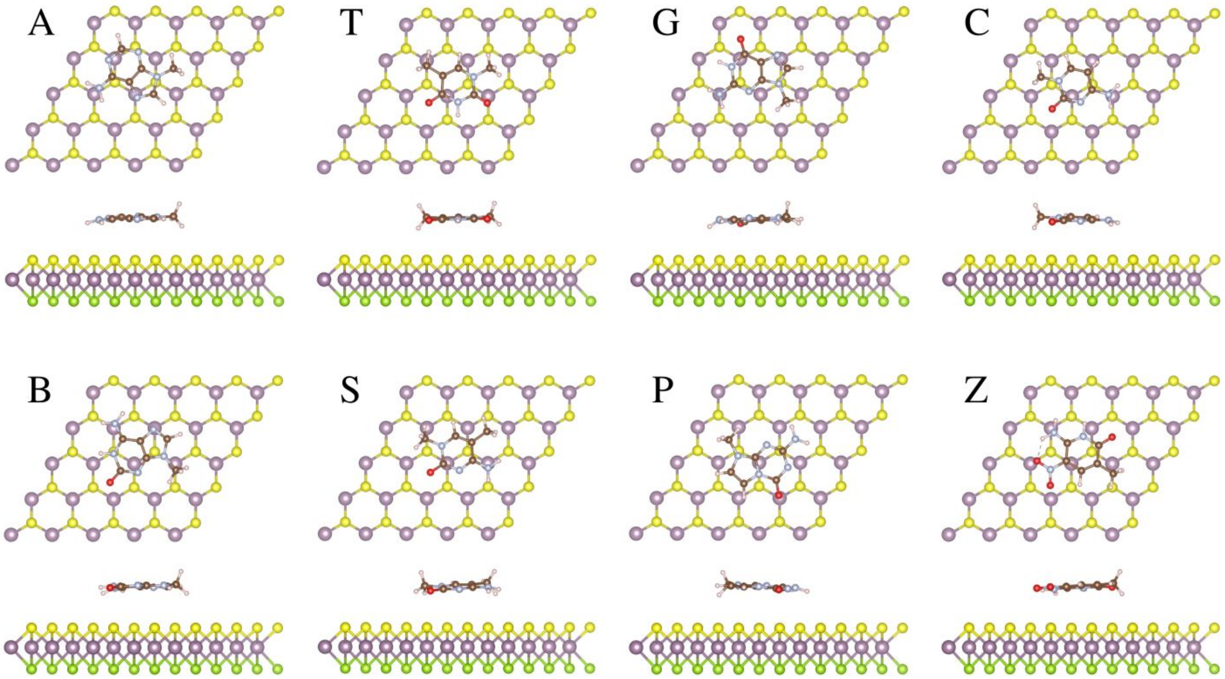

Figure S2. Top and side view of the lowest energy conformations of natural (A, T, G, C) and modified (B, S, P, Z) base molecules on the S side of the MoSSe monolayer. Mo, S, Se, C, N, O, and H atoms are represented by purple, yellow, green, brown, gray, red, and pink spheres, respectively.

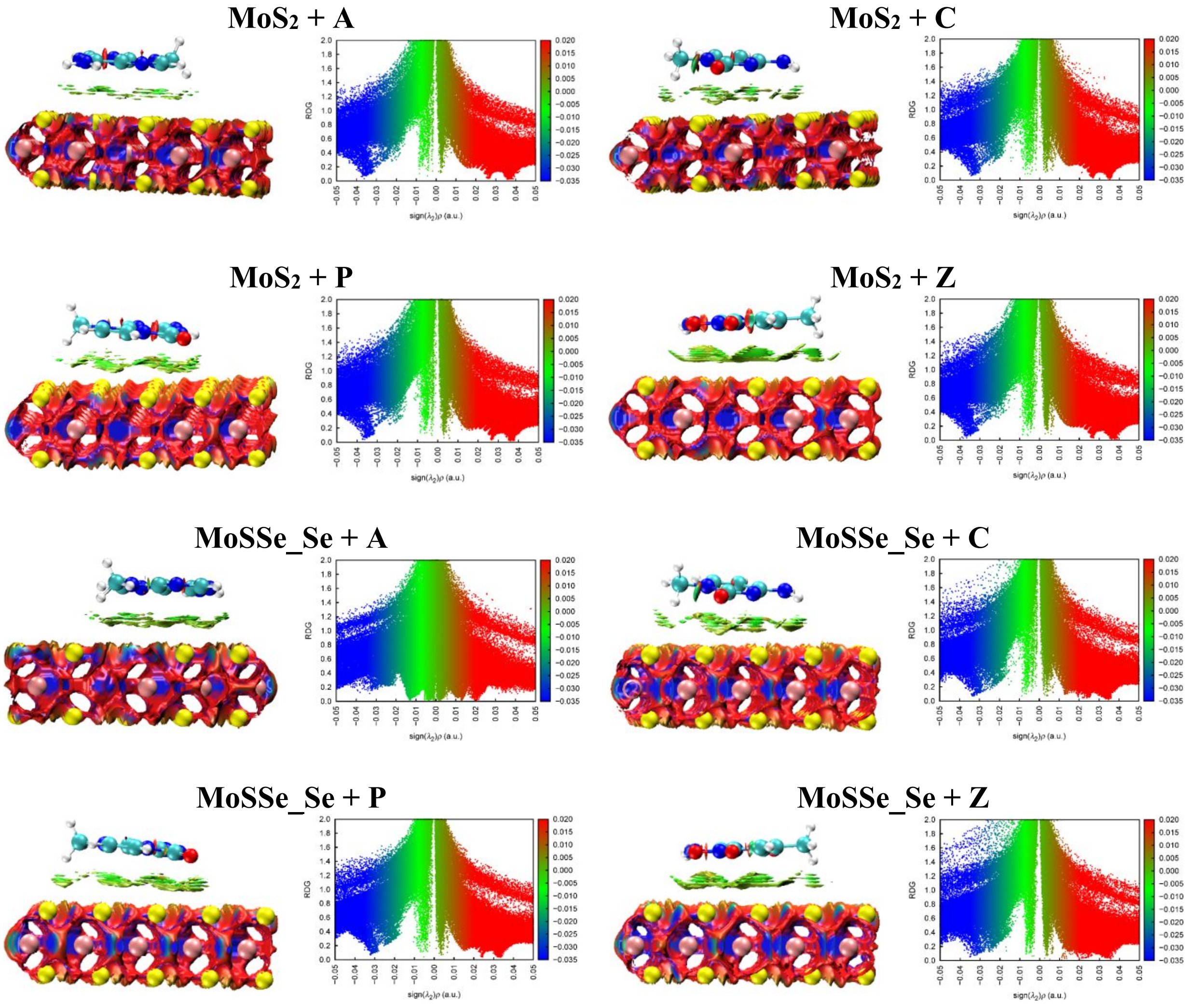

Figure S3: NCI surface and NCI plot illustrating the base-MoS_2/MoSSe_Se surface interaction.

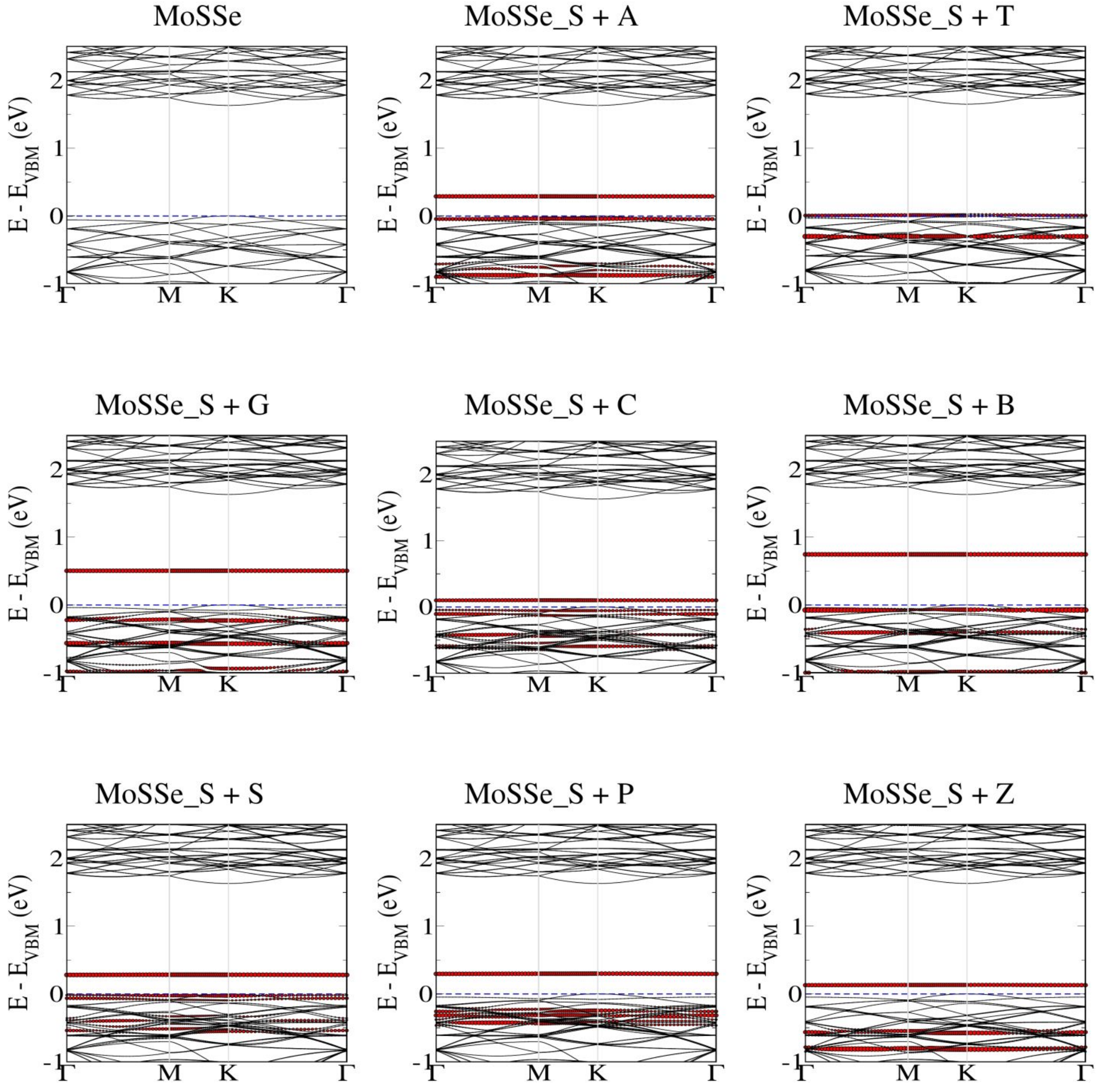

Figure S4. Electronic band structure of the natural (A, T, G, C) and modified (B, S, P, Z) base molecules on S side of MoSSe monolayer. Molecular contributions are highlighted in red color.

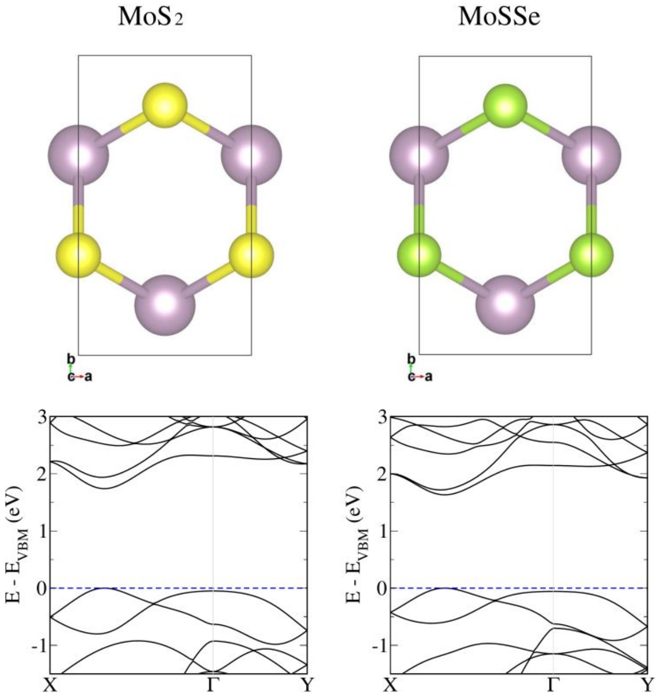

Figure S5. Atomic structure (top row) with electronic band structure (bottom row) of rectangular unit cell of MoS_2 and MoSSe monolayers. The Mo, S, and Se atoms are represented by purple, yellow and green color balls, respectively.

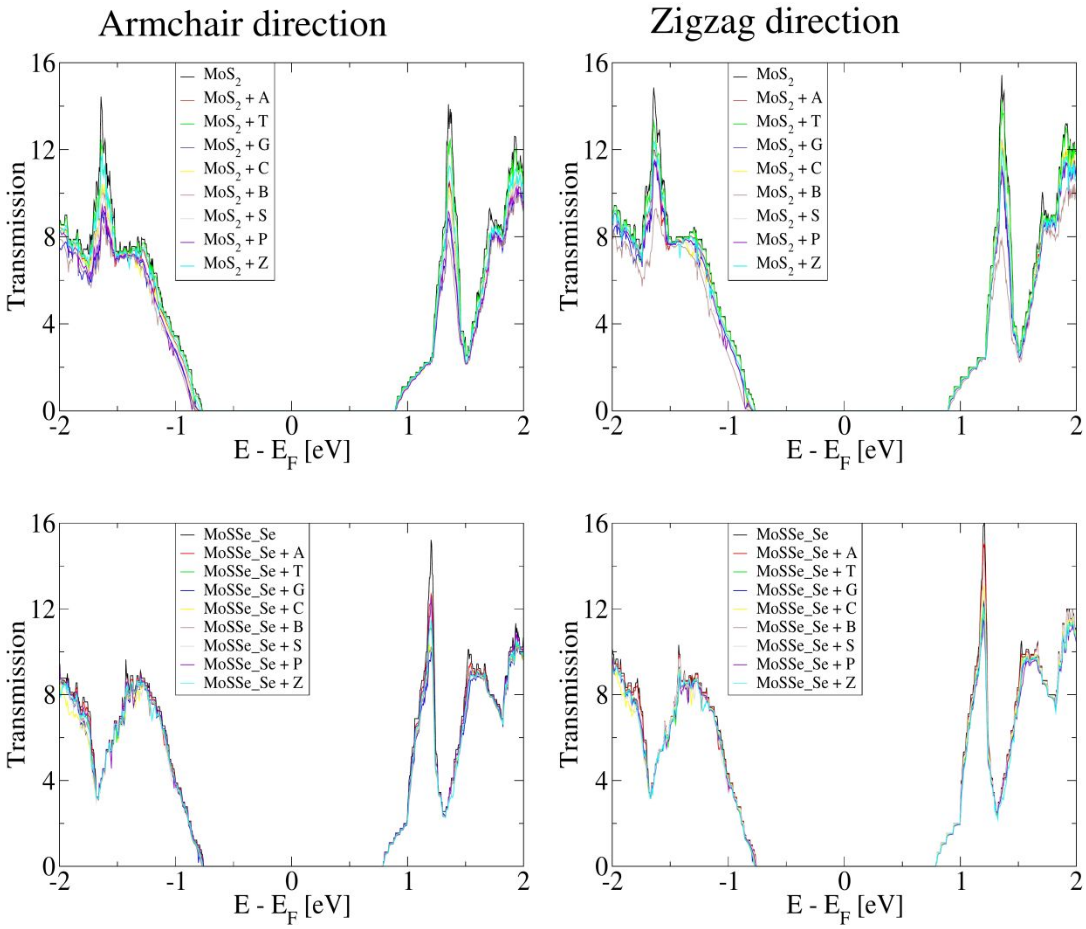

Figure S6. Transmission coefficient plots at applied zero bias voltage for MoS_2 and MoSSe (Se side) along armchair and zigzag direction without and with bases molecules.

References:

[1] Contreras-García, J.; Johnson, E. R.; Keinan, S.; Chaudret, R.; Piquemal, J. P.; Beratan, D. N.; and Yang, W.; et al. NCIPLOT: A program for plotting noncovalent interaction regions. *J. Chem. Theory Comput.,* **2011**, 7, 625–632.

[2] Frisch, M. J.; Trucks, G. W.; Schlegel, H. B.; Scuseria, G. E.; Robb, M. A.; Cheeseman, J. R.; Scalmani, G.; Barone, V.; Petersson, G. A.; Nakatsuji, H.; Li, X.; Caricato, M.; Marenich, A. V.; Bloino, J.; Janesko, B. G.; Gomperts, R.; Mennucci, B.; Hratchian, H. P.; Ortiz, J. V.; Izmaylov, A. F.; Sonnenberg, J. L.; Williams-Young, D.; Ding, F.; Lipparini, F.; Egidi, F.; Goings, J.; Peng, B.; Petrone, A.; Henderson, T.; Ranasinghe, D.; Zakrzewski, V. G.; Gao, J.; Rega, N.; Zheng, G.; Liang, W.; Hada, M.; Ehara, M.; Toyota, K.; Fukuda, R.; Hasegawa, J.; Ishida, M.; Nakajima, T.; Honda, Y.; Kitao, O.; Nakai, H.; Vreven, T.; Throssell, K.; Montgomery Jr., J. A.; Peralta, J. E.; Ogliaro, F.; Bearpark, M. J.; Heyd, J. J.; Brothers, E. N.; Kudin, K. N.; Staroverov, V. N.; Keith, T. A.; Kobayashi, R.; Normand, J.; Raghavachari, K.; Rendell, A. P.; Burant, J. C.; Iyengar, S. S.; Tomasi, J.; Cossi, M.; Millam, J. M.; Klene, M.; Adamo, C.; Cammi, R.; Ochterski, J. W.; Martin, R. L.; Morokuma, K.; Farkas, O.; Foresman, J. B.; Fox, D. J.; et al. Gaussian16, Revision C.01, Wallingford, CT, 2016.

[3] Lu, T.; and Chen, F.; et al. Multiwfn: A Multifunctional Wavefunction Analyzer, J.

Comp. Chem. **2012**, 33, 580–592.

[4] Humphrey, W.; Dalke, A.; and Schulten, K.; et al. VMD - Visual Molecular Dynamics. *J. Molec. Graphics* **1996**, 14.1, 33–38.
